# Undifferentiated Carcinoma with Osteoclast-like Giant Cells of the Pancreas: Molecular Genetic Analysis of 13 Cases

**DOI:** 10.3390/ijms25063285

**Published:** 2024-03-14

**Authors:** Jan Hrudka, Markéta Kalinová, Vanda Ciprová, Jana Moravcová, Radim Dvořák, Radoslav Matěj

**Affiliations:** 1 Department of Pathology, 3rd Faculty of Medicine, Charles University, University Hospital Kralovske Vinohrady, 100 34 Prague, Czech Republic; marketa.kalinova@fnkv.cz (M.K.); morj@ikem.cz (J.M.); radoslav.matej@ftn.cz (R.M.); 2 Central Laboratories, University Hospital Kralovske Vinohrady, 100 34 Prague, Czech Republic; 3 Department of Pathology, 1st Faculty of Medicine, Charles University, General University Hospital, 128 00 Prague, Czech Republic; vanda.ciprova@vfn.cz; 4 Clinical and Transplant Pathology Center, Institute for Clinical and Experimental Medicine, 140 21 Prague, Czech Republic; 5 Department of General Surgery, 3rd Faculty of Medicine, Charles University, University Hospital Kralovske Vinohrady, 100 34 Prague, Czech Republic; radim.dvorak@fnkv.cz; 6 Department of Pathology and Molecular Medicine, 3rd Faculty of Medicine, Charles University, Thomayer University Hospital, 140 59 Prague, Czech Republic

**Keywords:** pancreas, undifferentiated carcinoma with osteoclast-like giant cells, next-generation sequencing, DNA

## Abstract

Undifferentiated carcinoma with osteoclast-like giant cells (UCOGC) of the pancreas is a rare malignancy regarded as a subvariant of pancreatic ductal carcinoma (PDAC) characterized by variable prognosis. UCOGC shows a strikingly similar spectrum of oncogenic DNA mutations to PDAC. In the current work, we analyzed the landscape of somatic mutations in a set of 13 UCOGC cases via next-generation sequencing (NGS). We detected a spectrum of pathogenic or likely pathogenic mutations similar to those observed in PDAC following previously published results (10 *KRAS*, 9 *TP53*, 4 *CDKN2A*, and 1 *SMAD4*, *CIC*, *GNAS*, *APC*, *ATM*, *NF1*, *FBXW7*, *ATR*, and *FGFR3*). Our results support the theory that UCOGC is a variant of PDAC, despite its unique morphology; however, a UCOGC-specific genomic signature as well as predictive markers remain mainly unknown. Programmed death ligand 1 (PD-L1) status remains an important predictive marker based on previous studies.

## 1. Introduction

Undifferentiated carcinoma with osteoclast-like giant cells (UCOGC) of the pancreas is regarded as a rare variant of pancreatic ductal adenocarcinoma (PDAC), according to the recent WHO classification [1], comprising about 0.4% of pancreatic carcinomas [2]. The first UCOGC description was published in 1954 by Sommers et al. [3]. Fourteen years later, Rosai labeled the lesion a “carcinoma simulating giant cell tumor of bone” [4]. UCOGC is characterized by specific morphology and variable prognosis. The tumor consists of three cell types: (1) neoplastic cytokeratin-positive mononuclear cells with nuclear atypia and pleomorphism, (2) non-neoplastic mononuclear histiocytes, and (3) non-neoplastic osteoclast-like multinucleated giant cells, present in variable amount, often in the vicinity of hemorrhage and necrosis. In the case of characteristic morphology, UCOGC diagnosis is based on histopathological examination [5]. However, despite their different morphology, the DNA mutational profiles in UCOGCs show surprising similarities with those of conventional PDAC [6], including the frequent activation of *KRAS* and deactivation of *SMAD4*, *TP53*, and *CDKN2A* [7,8,9]. The carcinogenesis of PDAC encompasses a cascade of molecular events, including telomere shortening, activating mutations in *KRAS*, inactivating mutations or the epigenetic silencing of p16/*CDKN2A*, and inactivating mutations in *TP53* and *SMAD4*. These cumulative alterations drive the progression from pancreatic intraepithelial neoplasia (PanIn) formation to the development of invasive adenocarcinoma [10]. NGS studies have reinforced the significance of recurrent mutations in four pivotal driver genes (*KRAS*, *TP53*, *SMAD4*, *CDKN2A*), each of which is disrupted in over 50% of PDACs, underscoring their central role within core signaling pathways [11]. The whole-genome sequencing of PDAC revealed 93% *KRAS-*, 74% *TP53-*, 35% *CDKN2A-*, and 31% *SMAD4*-mutated cases [12].

From a clinical point of view, UCOGC is characterized by mostly poor prognosis [13,14], but there are reports describing “unexpected long survivors” with a better prognosis compared to patients with PDAC [2,15]. A recent meta-analysis involving 69 patients revealed that UCOGC exhibits a notably superior prognosis in contrast to both PDAC and undifferentiated carcinoma (UC) lacking osteoclastic giant cells [16]. Hence, it is necessary to differentiate UCOGC from UC histologically, owing to the substantial prognostic variance [17]. Luchini et al. demonstrated that a crucial determinant of prognosis is the coexistence of a PDAC within UCOGC. Their findings revealed a median overall survival of 36 months for pure UCOGC, contrasting with 15 months for UCOGC accompanied by PDAC [6]. In recent years, there have been few studies documenting significant programmed death ligand 1 (PD-L1) expression in undifferentiated pancreatic carcinoma compared to PDAC [18] and the aggressive behavior of UCOGCs expressing PD-L1 compared to PD-L1-negative UCOGCs [19,20]. The aim of our study was to perform a molecular genetic analysis by the next-generation sequencing (NGS) of thirteen UCOGC cases that were analyzed by immunohistochemistry in our previous study [20]. Of these previously published cases, one was excluded due to insufficient material amount, and one new case was acquired.

## 2. Results

We isolated DNA from thirteen formalin-fixed paraffin-embedded (FFPE) tissue samples, and after an integrity check (PCR amplification of gene segments sized 100, 200, 300, 400, and 600 bp), we detected only 100 bp PCR products indicating DNA degradation in four samples. In six samples, we detected control PCR products of sizes 100 and 200 bp, and only in three samples, we observed amplification products larger than 200 bp in the control. All thirteen FFPE samples were examined by the NGS panel, and in only three samples, we could not achieve sufficient coverage due to DNA degradation in the archival FFPE tissue material. These were the samples where the control amplification showed only 100 bp products. For these three samples, we conducted NGS analysis with parameters set at a minimum coverage of 50 and a minimum variant frequency of 25% (samples ID2, ID3, and ID9).

As part of the repetition and validation of the NGS panel, we re-examined two FFPE tissue samples (samples ID4 and ID10). We found the same variants in ID4 (*TP53*, *KRAS*, *CDKN2A*, and *SMAD4*). In sample ID10, we found identical mutations in the *TP53* and *CHEK2* genes. Distinct findings in the *ERBB2*, *PDGFRA*, and *MTOR* genes (variants of uncertain significance) are attributed to the presence of variants at low percentages (6–7%); these findings may also be because all cases involve larger deletions (88–117 bp) that might be less detectable using NGS chemistry with 2 × 75 v3 reads. We primarily employ the given NGS panel and subsequent analysis for the detection of SNVs/Indels.

**Figure 1:**
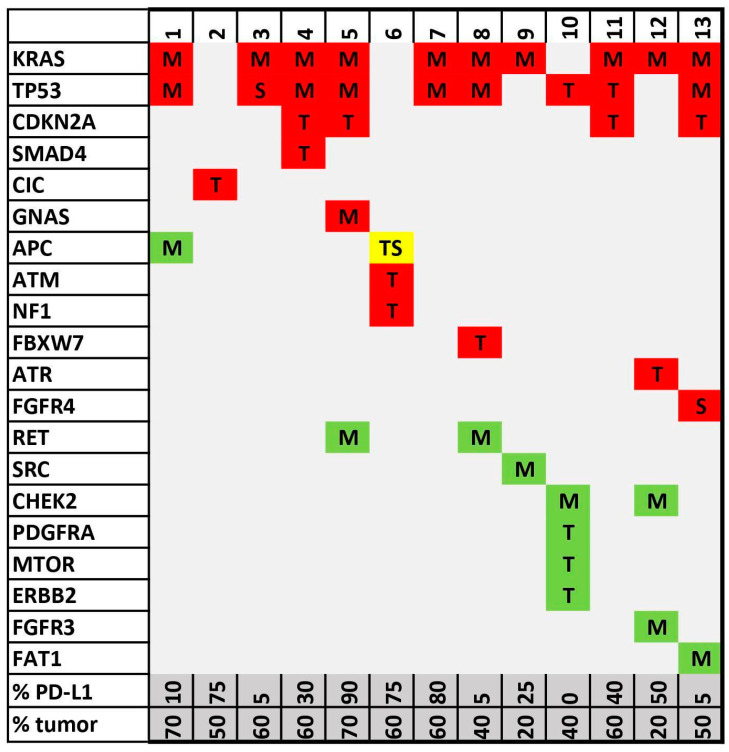
NGS oncoprint diagram shows detected alteration spectra in the analyzed sample set. Genes are sorted according to the number of detected alterations in the vertical columns and sample IDs in the horizontal rows. Alterations are indicated by a standard variant naming algorithm: T = truncating variant (including frameshift); M = oncogenic missense variant; S = oncogenic splicing variant. Red—pathogenic/likely pathogenic; green—variant of unknown significance (VUS); yellow—one pathogenic mutation and the other VUS. PD-L1 tumor proportion score (TPS) and percentage of tumor cells in NGS sample assessed by pathologist are presented in two bottom lines.

Using the NGS custom panel, we found the following pathogenic or likely pathogenic mutations: ten *KRAS*, nine *TP53*, four *CDKN2A*, and one *SMAD4*, *CIC*, *GNAS*, *APC*, *ATM*, *NF1*, *FBXW7*, *ATR*, and *FGFR4* (Figure 1). From the cohort, twelve cases were considered PD-L1-positive and one case PD-L1-negative, which does not allow meaningful statistical analysis.

The most frequent *KRAS* mutations were found in exon 2 codon 12, specifically 6× p.Gly12Asp, 3× p.Gly12Val, and 1× p.Gly12Arg.

All results with clinical–pathological parameters and PD-L1 expression profiles are listed in Table 1. Detailed NGS findings using VarSome Clinical software (version 11.7, Saphetor, Lausanne, Switzerland) are summarized in Supplementary Materials.

## 3. Discussion

Due to the rarity of UCOGC, only a few studies have focused on the genetic background of this peculiar neoplasia. The findings from genomic articles on UCOGC, including this study, are summarized in Table 2.

Luchini et al. performed the whole exome sequencing (WES) of eight UCOGC, which led to the identification of the same canonical oncogenic mutation (*KRAS*) and tumor suppressor gene alterations (*TP53*, *CDKN2A*, *SMAD4*) as reported in PDAC; the *KRAS* mutational spectrum documented by Luchini et al. in all examined UCOGC cases (4× p.Gly12Val, 3× p.Gly12Asp, and 1× p.Gly12Arg) [6] was very similar to this study (6x p.Gly12Asp, 3x p.Gly12Val, and 1× p.Gly12Arg). Imai et al. examined mutational profiles in three UCOGC cases, finding *KRAS* mutations in all cases, similar to our data (2× p.Gly12Asp and 1× p.Gly12Val) [21]. Deckard-Janatpour described *KRAS* mutations in five of six UCOGC cases and mutated p53 immunoprofiles in five of ten cases [22]. On the other hand, Lukáš et al. describe p53 and *KRAS* wild-type status in two UCOGC cases [23]. Analyzing four UCOGC cases and using a tissue microdissection technique, Sakai et al. found the *KRAS* mutation in both pleomorphic neoplastic UCOGC cells and epithelial cells of associated PDAC [24].

In this study, we used a custom panel focused on common solid tumor-related genes and examined a few more cases; to the best of our knowledge, this is the largest UCOGC cohort examined by NGS published to date. Our findings of ten *KRAS*, nine *TP53*, four *CDKN2A*, and one *SMAD4*, *CIC*, *GNAS*, *APC*, *ATM*, *NF1*, *FBXW7*, *ATR*, and *FGFR3* as pathogenic or likely pathogenic mutations within UCOGC mirrors the mutational spectrum described in PDAC (93% *KRAS*, 74% *TP53*, 35% *CDKN2A*, and 31% *SMAD4*) [12]. The identification of a similar spectrum of undifferentiated carcinoma (UDC) and UCOGC of the pancreas and PDAC led to the classification of these as variants of PDAC [1,25,26,27,28]. Interestingly, Luchini et al. found non-synonymous somatic missense mutations in serpin peptidase inhibitor clade A member (*SERPINA*) 3, melanoma-associated antigen (*MAGE*) B4, glioma-associated oncogene (*GLI*) *3*, multiple epidermal growth factor-like domains protein (*MEGF*) 8 (each mutated in two patients), and *TTN* (mutated in three tumors) [6]. Of note, we found pathogenic mutations *APC*, *ATM*, and *NF1* in one single UCOGC case—the aforementioned mutations have not been hitherto described in the literature. Yang discovered concomitant *KRAS* and *BRCA2* mutations in one UCOGC case using WES [29]; of note, 17% of PDAC have some “BRCAness” genetic signatures associated with homologous recombination deficiency (*BRCA2*, *BRCA1*, *PALB2*, *ATM*, and *RAD51*) [30]. In our dataset, there was one case with a pathogenic or likely pathogenic *ATM* mutation. Theoretically, patients with pancreatic cancer of any histological type showing signatures of “BRCAness” may benefit from poly-ADP-ribose polymerase (PARP) inhibitor (e.g., olaparib) biological therapy [30]. In our cohort, there was one case with an *FGFR3* alteration of unknown significance. In the FIGHT-101 clinical trial, one of five patients with pancreatic cancer showed a partial response to anti-FGFR1-3 treatment (pemigatinib); this case was positive for *FGFR2::USP33* fusion [31]. The study mentioned above delineates clinical outcomes across different tumors characterized by *FGFR* fusions/rearrangements and mutations. However, the therapeutic efficacy for the patients in our sample is uncertain, given that we only identified *FGFR3* variants of unknown significance. Although OncoKB indicates FDA approval for erdafitinib in *FGFR3*-mutated bladder cancer, there are currently no FDA-approved treatments tailored specifically to patients with *FGFR3* oncogenic mutant pancreatic cancer [32].

**Table 2:** Summary of genomic articles focused on UCOGC DNA mutational profiles obtained from FFPE tissue.

Authors	Year	UCOGC n=	Method	Mutated Genes (Pathogenic), Number of Cases
Present study	2024	13	Illumina MiSeq, NGS custom panel	10× *KRAS*, 9× *TP53*, 4× *CDKN2A*, 1× *SMAD4*, *CIC*, *GNAS*, *APC*, *ATM*, *NF1*, *FBXW7*, *ATR*, *FGFR3*
Yang et al. [27]	2020	1	Whole exome sequencing	1× *KRAS*, 1× *BRCA2*
Luchini et al. [6]	2017	8	Illumina HiSeq, whole exome sequencing	8× *KRAS*, 7× *TP53*, 3× *TTN*, 2× *CDKN2A*, *SERPINA3*, *MAGEB4*, *GLI3*, *MEGF8*, 1× *SMAD4*
Lukáš et al. [23]	2006	2	PCR	2× *KRAS* wild type, 2× *TP53* wild type
Sedivy et al. [28]	2005	1	PCR	1× *KRAS*
Sakai et al. [24]	2000	3	PCR	3× *KRAS*
Imai et al. [21]	1999	3	PCR	3× *KRAS*
Deckard-Janatpour et al. [22]	1998	6	PCR	5× *KRAS*
Westra et al. [25]	1998	5	PCR	4× *KRAS*
Hoorens et al. [26]	1998	1	PCR	1× *KRAS*

One case in our study displayed pathogenic *ATR* mutation, while the upregulation of the ATR pathway may play a role in resistance to PARP inhibitors in ovarian cancer cell lines. Patients with an *ATR*-mutated tumor profile may hypothetically benefit from anti-ATR treatment (berzosertib, ceralasertib). To date, there are ongoing clinical trials focused on anti-ATR combined with PARP inhibitors in advanced solid tumors [33]. Moreover, Sato et al. reported that *ATM*, *ATR* (each mutated in a single case in our cohort), and *CHK1* can drive PD-L1 expression [34]. Accordingly, ATR or CHK1 inhibition decreases PD-L1 expression and may facilitate the T-cell-mediated killing of the tumor cell [35,36], which may be of particular interest in UCOGC characterized by frequent PD-L1 expression. While the PARP inhibitor talazoparib in combination with enzalutamide is FDA approved for the treatment of select patients with ATR-mutant metastatic castration-resistant prostate cancer, no ATR-targeted therapy for pancreatic cancer was identified in the OncoKB database [32]. Interestingly, the single patient in our dataset has exhibited long recurrence-free survival without the need for specific therapy to date.

Like in conventional PDAC, *KRAS* and *TP53* were the most frequent alterations identified in our dataset. The therapeutic efficacy of adagrasib and sodagrasib has been reported for patients with *KRAS* p.Gly12Cys mutant pancreatic adenocarcinoma [32]. However, our dataset did not include any patients with *KRAS* p.Gly12Cys mutation; instead, we observed mutations only in p.Gly12Asp, p.Gly12Val, and p.Gly12Arg. Furthermore, the therapeutic impact of this treatment in UCOGC compared to PDAC remains a subject of debate.

There has been extensive and enduring research focused on restoring the physiological activity of the p53 protein. The objective is to maintain cell cycle homeostasis and programmed cell death, either through the induction of synthetic lethality in *TP53*-mutated cells or by revitalizing p53 functionality [37]. *TP53* transcription products emerge as promising targets for cancer therapy, given that *TP53* mutations are present in approximately 50% of malignant tumors and are absent in physiological tissues. The first substance identified for restoring the transcriptional activity of mutated p53, CP-31398, was documented in 1999 and demonstrated efficacy in murine experiments [38]. Subsequently, multiple drugs with similar mechanisms have been discovered. Over the past decade, numerous cell line tests and clinical trials involving anti-p53 treatments have been conducted, yielding equivocal clinical outcomes [39].

The analyzed cohort exhibits high variability in survival time, ranging from 0.1 to 171 months. The patient with the shortest survival succumbed to postoperative complications, and subsequent follow-up lacks sufficient information regarding tumor prognostics. Conversely, the patient with the longest survival had PD-L1-negative UCOGC, a condition associated with a relatively favorable prognosis, as previously reported after merging data from two studies [20]. A limitation of this study is the small cohort size, reflecting the overall rarity of this particular tumor type.

Despite several genetic studies on UCOGC, the knowledge of a specific genetic marker of this particular tumor is still insufficient; larger datasets are hard to obtain because of the rarity of this tumor. Diagnosis is often feasible histologically, but further research on potential therapy targets is needed. In recent years, PD-L1 has been discovered as frequently expressed in undifferentiated pancreatic carcinoma [18] and as a significant negative survival predictor in UCOGC [19,20], whilst the exceedingly rare PD-L1-negative UCOGCs display a good prognosis. Besides PD-L1 expression, an associated glandular (ductal) component has been described as an adverse prognostic factor [2]. Two cases of UCOGC that were treated successfully with anti-PD-L1 drugs were recently described; in one case, pembrolizumab therapy was administered due to positive PD-L1 immunohistochemistry [40], and in another case, due to high tumor mutational burden (TMB) [41]. An association between PD-L1 expression and p53 mutation has been described [19] but has not been confirmed by our group [20].

## 4. Materials and Methods

### 4.1. Sample Selection

A group of 13 patients (9 male, 4 female, age 50–76 years) with surgically treated or biopsied pancreatic UCOGC diagnosed histologically in three involved institutions between 2003 and 2019 was enrolled in the study. Among the cases, 10 specimens were pancreatectomies, 2 cases were metastasectomies (1 lymph node metastasis and 1 liver metastasis), and 1 case was a needle biopsy. Among the 10 pancreatectomy cases, 7 patients displayed regional lymph node metastasis at the time of surgery. In the 2 cases with metastasectomies, specimens from lymphadenectomy and liver metastasectomy during explorative laparotomy without pancreas resection were used. In the cohort, there were 8 pure UCOGCs, 3 UCOGCs mixed with PDAC, and 2 UCOGCs mixed with intraductal papillary mucinous neoplasm (IPMN) of the pancreas. No patient received immunotherapy, while some patients received adjuvant chemotherapy and radiotherapy. The assignments of specific diagnosis, stage, follow-up, immunohistochemistry, and NGS profile are listed in Table 1. Representative blocks with FFPE tumor tissue were used for both immunohistochemistry and molecular examination. Clinical data about survival and disease progression were collected.

### 4.2. Immunohistochemistry

**Figure 2:**
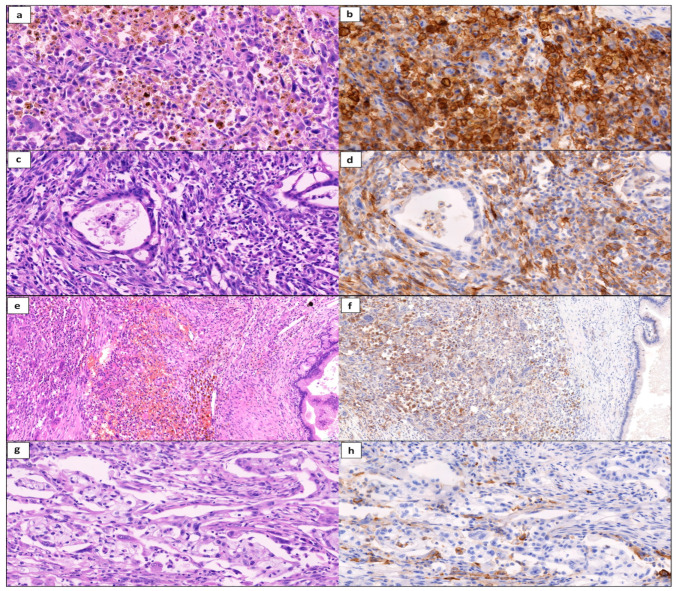
Scans of histology slides in undifferentiated carcinoma with osteoclast-like giant cells (UCOGC) of the pancreas and its variants, stained with hematoxylin and eosin and anti-PD-L1 antibody: (**a**,**b**) pure UCOGC with diffuse strong PD-L1 positivity (40×), (**c**,**d**) UCOGC combined with pancreatic ductal adenocarcinoma (PDAC), note the PD-L1-positive UCOGC and negative PDAC component (40×), (**e**,**f**) UCOGC combined with intraductal papillary mucinous neoplasm (IPMN), note the PD-L1-positive UCOGC and negative IPMN (20×), (**g**,**h**) PDAC component in combined UCOGC-PDAC with PD-L1 negativity, positivity only in macrophages (40×).

For immunohistochemistry, 4 μm thick tissue sections were stained using a Dako Autostainer Link 48 IHC Stainer w/ PT Link (Agilent Technologies, Santa Clara, CA, USA) using Dako PD-L1 IHC 22C3 pharmDx diagnostic kit (Dako, Agilent Technologies, Santa Clara, CA, USA) following the manufacturer’s instructions. The slides were counterstained with hematoxylin. Stained slides were dehydrated and covered in a xylene-based mounting medium. All immunohistochemical examinations were assessed using a microscope by two experienced routine pathologists (JH and RM, Figure 2). PD-L1 was considered positive when membranous staining occurred in > 1% of neoplastic cells as the tumor proportion score (TPS) following standard recommendations [42] as described in our previous publication [20]. In cases of discordant findings using a multihead microscope, a PD-L1-positive percentage was numbered manually using CaseViewer software (version 2.4, 3DHistech, Budapest, Hungary) in digital histology scan.

### 4.3. Next Generation Sequencing

For molecular analysis, FFPE tissue sections from representative UCOGC tissue were used. In the case of tumors mixed with PDAC or IPMN, only the UCOGC component was microdissected. The DNA was extracted from eight to ten 5 µm thick sections employing the QIAamp FFPE Tissue Kit (Qiagen, Hilden, Germany). The concentrations of DNA were measured using a Qubit 3 Fluorometer with the Qubit dsDNA BR Assay Kit (Invitrogen, Thermo Fisher Scientific, Wilmington, DE, USA). The integrity and amplifiability of DNA extracted from FFPE tissue were checked according to the Biomed-2 recommendation [43]. We designed a custom NGS panel (Agilent Technologies, Santa Clara, USA) to detect Single Nucleotide Variants (SNVs) and Indels in 73 genes associated with solid tumors. The NGS custom panel was designed to cover genes crucial in pancreatic carcinogenesis according to a previously published panel [44]. The preparation of the NGS libraries was performed using the protocol for Illumina paired-end multiplexed library preparation, which involved enzymatic fragmentation and the SureSelectXT Library Preparation and Capture System (SureSelect XT Target Enrichment, Agilent Technologies). The NGS library was sequenced on the MiSeq instrument using the 2 × 75 v3 kit (Illumina, San Diego, CA, USA). The resulting FASTQ data underwent quality control checks and trimming. Indexing, mapping, alignment, and classification were accomplished through Varsome Clinical software (version 11.7, Saphetor, Lausanne, Switzerland) [45], utilizing the hg38 reference genome. The minimum frequency of variants was set to 5%, minimum coverage 100 reads.

### 4.4. List of Genes in Custom NGS Panel

*AKT1*, *ALK*, *APC*, *ARAF*, *ARID1A*, *ARID1B*, *ATM*, *ATR*, *BRAF*, *BRCA1*, *BRCA2*, *CCND1*, *CDK12*, *CDKN2A*, *CDKN2B*, *CIC*, *CSF1R*, *CTNNB1*, *DDR2*, *EGFR*, *ERBB2*, *ESR1*, *FAT1*, *FBXW7*, *FGFR1*, *FGFR2*, *FGFR3*, *FGFR4*, *GATA3*, *GNA11*, *GNAQ*, *GNAS*, *H3-3A*, *H3C2*, *HDAC2*, *CHEK1*, *CHEK2*, *IDH1*, *IDH2*, *KEAP1*, *KIT*, *KRAS*, *MAP2K1*, *MDM2*, *MDM4*, *MET*, *MTOR*, *MYB*, *MYC*, *MYCN*, *NF1*, *NOTCH1*, *NRAS*, *NTRK1*, *NTRK2*, *NTRK3*, *PALB2*, *PDGFRA*, *PIK3CA*, *PIK3R1*, *POLE*, *PTEN*, *PTCH1*, *PTPN11*, *RB1*, *RET*, *ROS1*, *SMAD4*, *SMARCA4*, *SMARCB1*, *SRC*, *STK11*, *TP53.*

## 5. Conclusions

No somatic genetic aberrations were identified that were obviously associated with either patient prognosis or PD-L1 status. From the standpoint of this analysis, there appears to be a justification for conducting PD-L1 immunohistochemistry as a routine procedure in UCOGC cases, together with a detailed genetic analysis focused on oncogenic and druggable molecular genetic signatures in all pancreatic cancers, regardless of histological subtype. Further data are required to clarify any potential correlations between pathogenic mutations, histological subtypes, and prognosis in pancreatic cancer.

## Figures and Tables

**Table 1:** Summary of all examined variables.

ID	Diagnosis	Specimen	Tumor Size (mm)	pTNM Stage at Surgery	Death	Survival/Follow-Up Time (Months)	Adjuvant Therapy	% of Tumor Cells	PD-L1 Positivity of Tumor Cells	Pathogenic/ Likely Pathogenic	Uncertain Significance
1	UCOGC+PDAC	Resection	30 mm	T2N1	Yes	5.7	Chemotherapy	70%	10%	*KRAS*, *TP53*	*APC*
2	UCOGC	Resection	70 mm	T3N1	Yes	1.2	No	50%	75%	*CIC*	
3	UCOGC	Resection	90 mm	T3N0	Yes	8.6	Unknown	60%	5%	*KRAS*, *TP53*	
4	UCOGC	Resection	18 mm	T1cN0	No	56.4	Chemotherapy	60%	30%	*TP53*, *KRAS*, *CDKN2A*, *SMAD4*	
5	UCOGC	Resection	60 mm	T3N1	No	58.3	Chemotherapy	70%	90%	*TP53*, *KRAS*, *GNAS*, *CDKN2A*	*RET*
6	UCOGC	Lymph node metastasis	unknown	TXN1	Yes	4.4	Unknown	60%	75%	*APC*, *ATM*, *NF1*	*APC*
7	UCOGC+PDAC	Resection	110 mm	T3N1	Yes	5.6	Unknown	60%	80%	*KRAS*, *TP53*	
8	UCOGC	Resection	140 mm	T3NXM1	Yes	0.1	No	40%	5%	*TP53*, *KRAS*, *FBXW7*	*RET*
9	UCOGC	Liver metastasis	>100 mm	T4NXM1	Yes	3.6	Unknown	20%	25%	*KRAS*	*SRC*
10	UCOGC	Resection	35 mm	T2N0M1	Yes	171	Chemotherapy, radiotherapy	40%	0%	*TP53*	*CHEK2*, *PDGFRA/MTOR/ERBB*
11	UCOGC+IPMN	Resection	9 mm UCOGC, 60 mm IPMN	T1bN0	No	89	No	60%	40%	*KRAS*, *TP53* *CDKN2A*	
12	UCOGC+IPMN	Resection	5 mm UCOGC 70 mm IPMN	T1aN0	No	49	Chemotherapy	20%	50%	*KRAS*, *ATR*	*CHEK2*, *FGFR3*
13	UCOGC+PDAC	Needle biopsy UCOGC+PDAC	34 mm	T2N1	Unknown	Unknown	Unknown	50%	5%	*KRAS*, *TP53*, *CDKN2A*, *FGFR4*	*FAT1*

## Data Availability

All data supporting reported results can be found from the institutions of the authors or in public repositories.

## Supplementary Materials

The following supporting information can be downloaded at: https://www.mdpi.com/article/10.3390/ijms25063285/s1.
